# SUMO Chains Rule on Chromatin Occupancy

**DOI:** 10.3389/fcell.2019.00343

**Published:** 2020-01-10

**Authors:** Jan Keiten-Schmitz, Kathrin Schunck, Stefan Müller

**Affiliations:** Institute of Biochemistry II, Medical Faculty, Goethe University, Frankfurt, Germany

**Keywords:** RNF4, StUbL, SENP6, PolySUMOylation, SUMO chains

## Abstract

The dynamic and reversible post-translational modification of proteins and protein complexes with the ubiquitin-related SUMO modifier regulates a wide variety of nuclear functions, such as transcription, replication and DNA repair. SUMO can be attached as a monomer to its targets, but can also form polymeric SUMO chains. While monoSUMOylation is generally involved in the assembly of protein complexes, multi- or polySUMOylation may have very different consequences. The evolutionary conserved paradigmatic signaling process initiated by multi- or polySUMOylation is the SUMO-targeted Ubiquitin ligase (StUbL) pathway, where the presence of multiple SUMO moieties primes ubiquitylation by the mammalian E3 ubiquitin ligases RNF4 or RNF111, or the yeast Slx5/8 heterodimer. The mammalian SUMO chain-specific isopeptidases SENP6 or SENP7, or yeast Ulp2, counterbalance chain formation thereby limiting StUbL activity. Many facets of SUMO chain signaling are still incompletely understood, mainly because only a limited number of polySUMOylated substrates have been identified. Here we summarize recent work that revealed a highly interconnected network of candidate polySUMO modified proteins functioning in DNA damage response and chromatin organization. Based on these datasets and published work on distinct polySUMO-regulated processes we discuss overarching concepts in SUMO chain function. We propose an evolutionary conserved role of polySUMOylation in orchestrating chromatin dynamics and genome stability networks by balancing chromatin-residency of protein complexes. This concept will be exemplified in processes, such as centromere/kinetochore organization, sister chromatid cohesion, DNA repair and replication.

## The Sumo Pathway and Its Intersection With Ubiquitin

Post-translational modification with the ubiquitin-related modifier SUMO provides a rapid and reversible way to control protein functions. Lower eukaryotes, such as *Saccharomyces cerevisiae* express a single SUMO form, also known as Smt3, while in humans three conjugatable SUMO paralogs (SUMO1, SUMO2, and SUMO3) are found (Flotho and Melchior, 2013; Cappadocia and Lima, 2018). At the amino acid level SUMO1 is 50% identical to SUMO2/3, which differ in only two amino acid residues. Conjugation of SUMO to lysine residues of targets proceeds via a multi-step enzymatic pathway involving a dimeric E1 activating enzyme (SAE1/SAE2), an E2 conjugating enzyme (Ubc9) and a relatively small set of SUMO E3 ligases. Attachment of SUMO generally modulates protein-protein interactions through binding of SUMO conjugates to interaction partners harboring specific SUMO interaction motifs, termed SIMs (Raman et al., 2013; Husnjak et al., 2016). Multiple SUMO-SIM mediated protein-protein interactions are commonly involved in the assembly of larger protein complexes. Much like ubiquitin, SUMO can be attached as a monomer on single or multiple lysine sites of a target protein generating mono- or multiSUMOylated proteins. Additionally, SUMO can also form polymeric chains through the attachment of one SUMO molecule to internal lysine residues of another SUMO moiety. SUMO chains are typically induced in response to cellular stress and preferentially assemble via lysine residue 11 in SUMO2/3 (Ulrich, 2008; Vertegaal, 2010). However, alternatively linked non-canonical SUMO2/3 chains, mixed SUMO1-SUMO2/3 chains as well as SUMO2/3 chains capped with SUMO1, have been found (Matic et al., 2008; Gartner et al., 2018; Sriramachandran et al., 2019). One signaling process initiated by SUMO chains is the SUMO-targeted Ubiquitin ligase (StUbL) pathway. In this evolutionary conserved pathway, poly- (or multi-) SUMOylated proteins are recognized by E3 ubiquitin ligases that contain specific binding modules for these structures. The best-characterized StUbLs are the budding yeast Slx5/Slx8 heterodimer and the mammalian RING-type ubiquitin ligases RNF4 and RNF111 (Sriramachandran and Dohmen, 2014; Kumar and Sabapathy, 2019). They all contain poly- or multi-SUMO binding modules and catalyze either non-proteolytic or proteolytic ubiquitylation of proteins modified by multiple SUMO moieties. Depending on the cooperating ubiquitin E2 enzyme, RNF4 and RNF111 can mediate either K63- or K48 ubiquitylation. RNF4 also synthesizes hybrid SUMO-ubiquitin chains by ubiquitylating lysine residues on SUMO (Guzzo et al., 2012). Similarly to other PTMs SUMOylation is a reversible and dynamic modification. Deconjugation of SUMO from targets is catalyzed by SUMO-specific isopeptidases. The best-characterized isopeptidases belong to the ULP/SENP family of cysteine proteases, which share a conserved catalytic domain (Hickey et al., 2012; Kunz et al., 2018). In *S. cerevisiae*, Ulp1 and Ulp2 act as SUMO deconjugases, while human cells express six SENP family members. SENP1, SENP2, SENP3 and SENP5 are evolutionary derived from the Ulp1 branch, while SENP6 and SENP7 are related to the Ulp2 subtype. Ulp2, as well as SENP6 and SENP7 preferentially act on SUMO chains thereby countering the StUbL pathway. While SENP6 and SENP7 antagonize the StUbL pathway by limiting SUMO chain formation, two deubiquitylating enzymes, namely USP7 and USP11, possibly counter RNF4 signaling by catalyzing the removal of ubiquitin from SUMO2 polymers (Hendriks et al., 2015; Lecona and Fernandez-Capetillo, 2016; Lecona et al., 2016).

**FIGURE 1:**
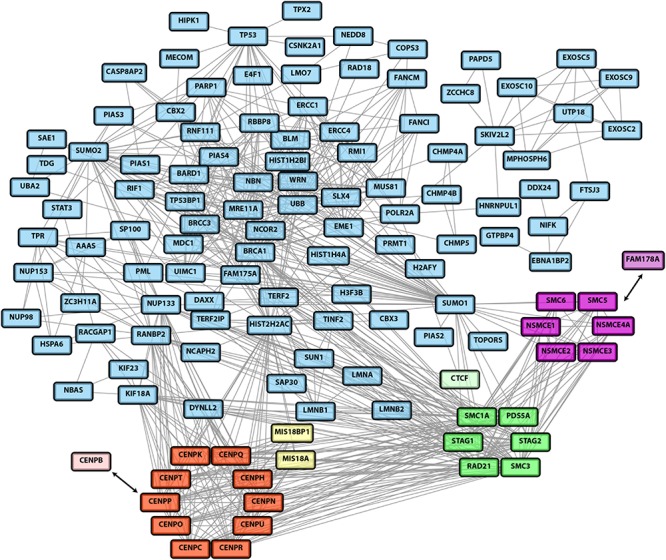
A network of SENP6 targets identified by two recent unbiased proteomic studies (Liebelt et al., 2019; Wagner et al., 2019). Candidate SENP6 targets identified in both studies were combined into a single dataset. This dataset was then used for the generation of a network using the STRING database (version 11.0, https://string-db.org). Only highest confidence interactions (interaction score > 0.9) were considered. Experiments and databases were used as interaction sources. Disconnected nodes were removed from the network. The network data was imported into Cytoscape. The core components of the constitutive centromere associated network (CCAN) are highlighted as red subcluster. The associated CENP-B and the CENP-A targeting factors Mis18A/Mis18BP1 are depicted in light red and yellow, respectively. The cohesion complex is represented by the green subcluster (with the CTCF targeting factor in light green) and the SMC5/6 complex is highlighted in magenta. The SMC5/6 recruitment factor FAM178A/Slf2 is shown in pink.

Despite these mechanistic insights, cellular signaling by SUMO chains is still incompletely understood. This is mainly due to the fact that so far the cellular substrates undergoing dynamic polySUMOylation were not well defined. To fill this gap, recent unbiased proteomic screens have focused on the identification of targets for the chain-specific isopeptidase SENP6 (Liebelt et al., 2019; Wagner et al., 2019). To this end alterations in SUMO conjugation were determined following inactivation of SENP6 by siRNA- or shRNA-mediated knock-down. Even though some changes might be indirect, these studies revealed a comprehensive list of candidate substrates of SUMO chain modification. Based on these datasets and other published work we give an overview of cellular networks that are controlled by polySUMOylation. The integrated proteomics dataset of candidate SENP6 targets revealed a highly interconnected network of proteins functioning in DNA damage response and DNA repair networks as well as chromatin organization. Importantly, SENP6 seems to limit SUMO chain formation of protein groups that are part of larger protein assemblies (Figure 1). This is exemplified for the constitutive centromere-associated network (CCAN) (Figure 1, red subcluster) and SMC (structural maintenance of chromosomes) complexes, such as the SMC1/3 cohesin complex (Figure 1, green subcluster) and the SMC5/6 complex (Figure 1, magenta subcluster).

## Sumo Chains as Organizers of Kinetochores/Centromeres

**FIGURE 2:**
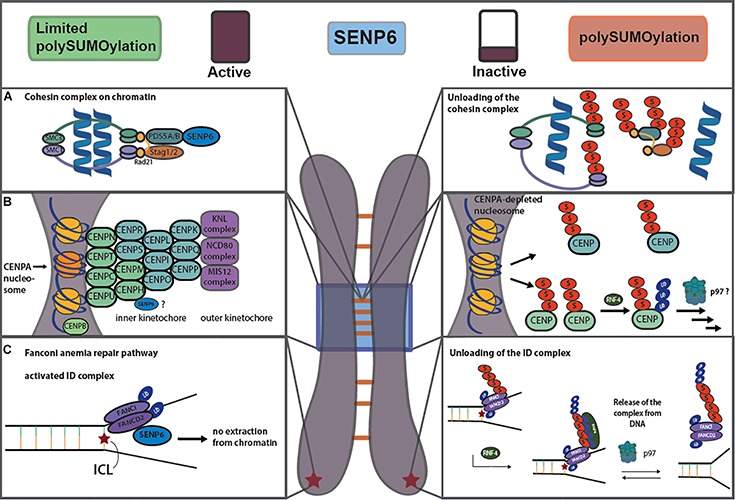
Balancing SUMO chain formation controls chromatin residency of the cohesion complex **(A)**, the centromere network **(B)** and the FA repair pathway **(C)**. For details see text.

A first hint for a role of SUMO, and in particular SUMO chains, in centromere organization was provided by genetic data from yeast. In fact, both Smt3 and Ulp2/Smt4 were initially identified in a high copy suppressor screen of a temperature sensitive mutation in the centromeric Mif2 protein, the yeast ortholog of the mammalian inner kinetochore protein CENP-C (Meluh and Koshland, 1995). Subsequent work in vertebrates indeed revealed that the restriction of SUMO chain formation by SENP6, the human ortholog Ulp2, is essential for the proper assembly of kinetochore/centromere structures. Dasso and co-workers demonstrated that SENP6 depletion enhanced SUMOylation and proteasomal degradation of CENP-I, thereby preventing deposition of the CENP-H/I/K subcomplex on kinetochores (Mukhopadhyay et al., 2010; Mukhopadhyay and Dasso, 2010). Co-depletion of the StUbL RNF4 together with SENP6 at least partially restored these defects, suggesting that RNF4 and SENP6 function antagonistically in this context. Indeed, it was further demonstrated that in the absence of SENP6 the polySUMOylated CENP-H/I/K complex is targeted by RNF4 for ubiquitylation and proteasomal degradation. The authors concluded that this leads to the loss of the CENP-H/I/K complex from kinetochores thereby causing defects in spindle assembly and mitotic progression. The CENP-H/I/K subcomplex is part of the constitutive centromere associated network (CCAN), which localizes to the centromere throughout the cell cycle and bridges the histone H3 variant CENP-A at centromeric chromatin to the microtubule-binding machinery (Figure 2B). Two recent unbiased proteomic screens now confirmed that SENP6 not only counters SUMO chain formation on CENP-I, but also on CENP-H and CENP-K. Moreover, it appears that multiple subunits within all other CCAN subcomplexes undergo unrestricted polySUMOylation in the absence of SENP6 (Liebelt et al., 2019; Wagner et al., 2019). Importantly, in cells lacking SENP6 polySUMOylation of these CCAN components coincides with their loss from centromeres (Liebelt et al., 2019). Surprisingly, polySUMOylation did not trigger their ubiquitylation and proteasome dependent degradation leading to the conclusion that polySUMO chain formation on CCAN proteins *per se* rather than subsequent RNF4 mediated ubiquitylation affects their proper deposition at centromeres (Figure 2B). Notably, however, the canonical RNF4-SENP6 StUbL pathway is critically involved in centromere architecture by controlling the centromeric deposition and/or maintenance of the master organizer CENP-A (Fu et al., 2019; Liebelt et al., 2019). This is not mediated by SUMOylation of CENP-A itself, but by RNF4 regulating the stability of the CENP-A recruitment factor Mis18BP1 (Fu et al., 2019; Liebelt et al., 2019). Noteworthy, in the yeast *S. cerevisiae* the CENP-A ortholog Cse4 is targeted for degradation by the Slx5/8 StUbL thereby preventing its mislocalization to euchromatin (Ohkuni et al., 2016). In mammalian cells, CENP-B dynamics at centromeres is also likely directly regulated by SUMO-primed RNF4-dependent proteasomal degradation (Maalouf et al., 2018), which is consistent with the identification of CENP-B as a major SENP6 target in proteomic studies (Liebelt et al., 2019; Wagner et al., 2019). Importantly, kinetochore-association of the motor protein CENP-E was also shown to depend on SUMO homeostasis at centromers (Zhang et al., 2008).

Altogether these data clearly indicate that the global architecture of centromere/kinetochores largely depends on the coordinated formation and editing of SUMO chains. Conclusive evidence defines SENP6 as the essential chain-limiting activity in this process and underscores a main role of SENP6 in protecting at least a subset of centromeric proteins from entering the StUbL pathway. Why only a subset of polySUMOylated centromeric proteins are channeled into this pathway and how the proposed polySUMO-mediated, but ubiquitin-independent impairment of centromeric CCAN deposition occurs are future key questions.

## Sumo Chains as Regulators of SMC Complexes

Structural maintenance of chromosomes complexes consist of multi-subunit annular structures that encircle DNA molecules and function in the organization and compaction of chromosomes. In eukaryotes there are three distinct SMC complexes, cohesin (SMC1/3), condensin (SMC2/4) and the SMC5/6 complex (Haering and Gruber, 2016). These evolutionary conserved complexes have central functions during chromosome segregation and genome maintenance. All SMC complexes share a common ring-like architecture comprising two SMC proteins that are connected to each other via a kleisin subunit. Additional factors typically associated with the kleisin subunit exhibit regulatory functions, in particular in chromatin loading or release of the complexes. Genetic and biochemical data from both lower and higher eukaryotes suggest that SUMO chain formation on SMC complexes is a critical determinant for their chromatin association.

The cohesion core complex is composed of the SMC1-SMC3 scaffold and the kleisin Rad21, which is associated with the HEAT repeat proteins STAG1 or STAG2 and PDS5A or PDS5B (Haering and Gruber, 2016). A main function of the cohesion ring in cycling cells is to hold sister chromatids together after DNA replication in S phase. In double-strand break (DSB) repair, the cohesin complex promotes homologous recombination in conjunction with the SMC5/6 complex (see below) by maintaining sister chromatids in close proximity. Cohesins also control gene expression by generating DNA loops that juxtapose enhancer and promoter elements. This particular function likely involves the CTCF protein as a cohesin targeting factor that tethers cohesins to specific CTCF binding sites. Loading and maintenance of cohesin in interphase involves the Rad21-associated PDS5A/B protein, although the underlying mechanism is not entirely clear. Data in both higher and lower eukaryotes now suggest that PDS5 at least partly functions by recruitment of a SUMO chain-editing activity to the cohesin core complex (Figure 2A) (Baldwin et al., 2009; Wagner et al., 2019). We detected an association of chromatin-associated PDS5 with SENP6 and found that SENP6 limits the polySUMOylation of PDS5 and all cohesin core components (Wagner et al., 2019). Lack of SENP6 affects sister chromatid cohesion and goes along with reduced chromatin association of RAD21 and STAG2 again linking unbalanced SUMOylation with perturbations in proper chromatin-residency and/or degradation (Wagner et al., 2019). Notably, in budding yeast, Ulp2 also controls SUMOylation of Pds5, and Ulp2 mutants are as well defective in cohesin maintenance (D’ambrosio and Lavoie, 2014). Moreover, in Pds5 mutants the yeast Rad21 ortholog is degraded by the StUbL pathway. Finally, in fission yeast SUMOylation of the SMC1/SMC3 orthologues Pms1 and Pms3 was specifically increased in a Slx8 mutant (Kohler et al., 2015). Notably, upon exposure of cells to DNA damaging agents cohesin subunits are SUMOylated by the SMC5/6-associated E3 ligase NSMCE2/MMS21 in yeast and mammals, and SUMOylation was reported to promote DSB repair through homologous recombination (McAleenan et al., 2012; Wu et al., 2012). Based on these findings we hypothesize that an initial monoSUMOylation event on cohesins promotes the establishment of cohesion, while their subsequent multi- or polySUMO-induced ubiquitylation promotes the dissolution of cohesion. SENP6/Ulp2 association with cohesins is therefore needed to keep cohesion hypersumoylation in check until dissolution should happen (Figure 2A). A key question in this yet speculative model remains how SENP6/Ulp2 targeting and/or activity is controlled. Given that the Plk1 ortholog Cdc5 interacts with yeast Ulp2 and opposes Ulp2 functions in centromeric cohesion, it is tempting to speculate that the phosphorylation status of Ulp2 determines its activity or localization (Baldwin et al., 2009). Investigating a potential phospho-dependent regulation of SENP6 is as well an attractive aspect for future studies.

Importantly, polySUMOylation of the two cohesin-related SMC complexes, condensin and SMC5/6 is also controlled by SENP6. Condensins promote chromatin compaction to prepare for chromosome segregation during mitosis. In humans two condensin complexes are found (Haering and Gruber, 2016). Condensin II resides in the cell nucleus during interphase and controls the early stage of chromosome condensation, whereas condensin I associates with chromosomes after nuclear envelope breakdown at the end of prophase. In both complexes the DNA encircling ring is formed by SMC2-SMC4 and the kleisin CAPH (Condensin I) or CAPH2 (Condensin II). In the condensin I complex CAPH is associated with the HEAT repeat proteins CAPD2 and CAPG, while CAPH2 in condensin II complexes is bound to CAPD3 and CAPG2. We detected a physical association of SENP6 with the CAPH2, CAPG2 subunits of condensin II and observed a strong increase in SUMOylation of the kleisin subunit CAPH2 in the absence of SENP6 (Wagner et al., 2019). The functional consequence of CAPH2 hyperSUMOylation in mammalian cells is not yet clear. However, deletion of Ulp2 in budding yeast affects is proper targeting to the rDNA locus (Strunnikov et al., 2001). Moreover, in fission yeast, condensin subunits were identified as Slx5/8 substrates (Kohler et al., 2015). Altogether these data are consistent with the idea that SENP6 is a crucial regulator of cohesion and condensin function by controlling their chromatin residency in conjunction with the RNF4 pathway. This concept can possibly be expanded to the SMC5-SMC6 complex, which is crucial for repair of DNA DSB and for replication stress tolerance. In the SMC5/6 complex the kleisin NSMCE4 forms a ring structure together with SMC5-SMC6 (Haering and Gruber, 2016). Among the regulatory factors associated with the complex are the ubiquitin-ligase NSMCE1 and the SUMO ligase NSMCE2, alias MMS21, and NSMCE3. MS data in SENP6 depleted cells show an enhanced SUMOylation of the core complex (SMC5, SMC6, NSMCE4) as well as NSCME1-3 (Liebelt et al., 2019). Moreover, NSMCE2 levels are strongly downregulated under these conditions indicating that polySUMOylation channels it into the RNF4 pathway (Wagner et al., 2019). This is consistent with proteomic data identifying NSMCE2 as a *bona fide* RNF4 substrate (Kumar et al., 2017). To better understand these processes future experiments should focus on the regulation of SENP6 during cell cycle progression and in response to DNA damage.

## Sumo Chains in DNA Repair and the DNA Damage Response (DDR)

In both lower and higher eukaryotes StUBLs contribute to the maintainance of genome stability (Heideker et al., 2009; Kumar and Sabapathy, 2019). A recurrent theme in DDR pathways is the SUMO-regulated turnover of repair factors at sites of DNA damage. SUMO-primed ubiquitylation appears to have a key role in extraction or clearance of DNA repair factors from chromatin. One well established example for this process has been delineated in the Fanconi anemia (FA) repair pathway (Gibbs-Seymour et al., 2015; Xie et al., 2015; Figure 2C). The canonical function of the FA pathway is the repair of DNA inter-strand cross-links (ICLs) that, if unrepaired, are prone to convert to double strand breaks. In the FA pathway, a network of repair factors cooperates to preserve genomic integrity by stabilizing replication forks, and by alleviating replication stress resulting from ICLs. FANC proteins can been subdivided into three groups, where group I comprises the FANC core complex that functions as a ubiquitin ligase monoubiquitylating the group II proteins FANCI and FANCD2, known as the ID complex. This monoubiquitylation is required for ID localization to the lesion and the subsequent coordination of the repair by group III proteins. Repair involves the recruitment of structure-specific endonucleases and the HR repair machinery. Importantly, data by Mailand and co-workers provide evidence that SUMO-primed K48 and K63-linked ubiquitylation by RNF4 facilitates the removal of the activated ID complex from the sites of DNA lesions (Gibbs-Seymour et al., 2015). For unloading RNF4 cooperates with the p97 segregase and its adaptor protein DVC1/Spartan. SENP6 antagonizes this pathway by limiting SUMO-chain formation on FANCI, most likely at the sites of DNA damage. In accordance with this data, the unbiased proteomics screens for SENP6 targets and binding proteins confirmed the physical association of SENP6 with the ID complex as well as increased SUMOylation of FANCI in the absence of SENP6 (Wagner et al., 2019). Moreover, in SENP6 depleted cells FANCD2 protein levels were strongly reduced indicating that in cells lacking SENP6, FANCD2 is not only extracted from chromatin but subsequently degraded by the StUbL pathway (Wagner et al., 2019). Altogether these data support the model that polySUMOylation in conjunction with RNF4, limits the dosage of activated ID complex at DNA lesions. It has been proposed that this regulatory circuit helps to avoid the prolonged, potentially dangerous localization of the structure-specific endonucleases to the chromatin (Gibbs-Seymour et al., 2015). Notably, SENP6 substrate profiling revealed that components of the endonuclease complexes itself, such as SLX4, ERCC1 or ERCC4 are as well targets of SENP6 and SLX4 is a *bona fide* RNF4 target (Kumar et al., 2017; Liebelt et al., 2019; Wagner et al., 2019). This possibly indicates that the StUbL pathway clears ID and endonuclease complexes from the DNA damage sites in a concerted action. In support of a coordinated clearance of the FA machinery FANCA, a component of the core complex, was also shown to be targeted for degradation by the SUMO-RNF4 pathway (Xie et al., 2015). The general concept of a polySUMO-primed StUbL-dependent eviction of protein complexes from chromatin appears to be a more widespread mechanism in DNA repair pathways. For example, in homologous recombination (HR) RNF4 controls the turnover of the replication protein A (RPA) at DNA damage sites (Galanty et al., 2012; Yin et al., 2012). In RNF4-depleted cells RPA is not properly replaced by the HR factor RAD51. SUMO-StUbL mediated extraction of repair factors is not limited to HR. In nucleotide excision repair (NER) the StUbL RNF111 promotes K63-linked ubiquitylation of the SUMOylated XPC repair factor, thereby promoting the release of XPC from damaged DNA after NER initiation (van Cuijk et al., 2015).

## Sumo Chains at Replisomes

Several lines of evidence point to a regulatory role of SUMO chains and the StUbL pathway in unperturbed DNA replication and under replication stress. A recurrent theme is again that the extent of SUMO- or Ub-SUMO chains governs the association of the replication machinery with chromatin. This has been very recently nicely exemplified for the budding yeast Dbf4-Cdc7 kinase complex, which mediates DNA replication initiation by phosphorylating the replicative MCM helicase complex (Psakhye et al., 2019). SUMO chains prime the replication engaged Dbf4-Cdc7 for Slx5/8-mediated degradation. Ulp2, which is directly associated with Dbf4, protects the complex from ubiquitylation thereby safeguarding replication initiation. This concept was expanded by the identification of additional factors, including the MCM helicase itself, as SUMO-chain-modified degradation-prone substrates of Ulp2 and Slx5/Slx8. The authors therefore propose SUMO-chain/Ulp2-protease-regulated proteasomal degradation as a mechanism that times the availability of functionally engaged SUMO-modified protein pools during replication.

Limiting the formation of Ub-SUMO conjugates at replisomes is also an important mechanism that safeguards replication progression in mammalian cells. USP7 was identified as a DUB that removes ubiquitin from SUMO. Intriguingly, upon USP7 inhibition, SUMOylated proteins are collectively displaced from the replisome, which fully abrogates DNA replication, both by limiting fork progression and the firing of new origins (Lecona and Fernandez-Capetillo, 2016; Lecona et al., 2016).

## Concluding Remarks

Altogether, the above-mentioned data strongly support a general role of polySUMOylation and the StUbL pathway in controlling the chromatin association of proteins and protein complexes. This also implies that mono- and polySUMOylation of the same protein or protein complex may have fundamentally different consequences. MonoSUMOylation can facilitate the assembly of DNA-associated protein complexes by fostering SUMO-SIM dependent complex formations. PolySUMOylation in turn primes the complexes for StUbL-mediated proteolytic or non-proteolytic ubiquitylation and displacement from chromatin in conjunction with the p97 machinery. DNA can directly trigger SUMOylation, as exemplified by DNA-dependent activation of the NSMCE2/Mms21 ligase in the SMC5/6 complex (Varejao et al., 2018). A key function of chain-trimming SUMO isopeptidases would therefore be to protect the complexes from polySUMOylation until release should occur. In line with this idea it has been proposed that SENPs are required to restrict an “over before it has begun” repair response (Garvin et al., 2019). Consistent with this scenario SENP6 localizes to sites of DNA damage in response to DNA damaging stimuli. StUBL-mediated clearance of protein complexes is likely not limited to replication or DNA repair processes, but seems as well play a role in promoter clearance during transcription (Martin et al., 2009; Rosonina et al., 2012; Ng et al., 2015; Akhter and Rosonina, 2016). However, the polySUMO-StUbL system does not always act in unloading chromatin-associated complexes, but can also prevent deposition to chromatin by acting on the soluble nucleoplasmic fraction of distinct complexes. This was initially shown for the CENH/I/K complex, which is degraded by RNF4 in S phase, and is now also exemplified on the CENP-A loading factor M18BP1 (Fu et al., 2019; Liebelt et al., 2019). Since SENP6 was not detected at centromeres in mitotic or in interphase cells it is likely that in these cases SENP6 limits hypersumoylation of centromere/kinetochore organizers in the nucleoplasmic fraction. How hyperSUMOylation alone without subsequent ubiquitylation controls chromatin residency of proteins in mammalian cells is unclear. Importantly, however, in both *S. pombe* and *S. cerevisiae* it has been shown that Cdc48/p97, in conjunction with its cofactor Ufd1, is targeted to SUMOylated proteins (Nie et al., 2012; Bergink et al., 2013). Moreover, Cdc48/p97 displaces the Rad52-Rad51 repair complex from chromatin in a SUMO-mediated, but ubiquitin-independent process (Bergink et al., 2013). Future work needs to uncover whether distinct p97 cofactors in mammalian cells are also solely dependent on polySUMO chains rather than ubiquitylation.

## Author Contributions

JK-S and KS designed and prepared the figures and corrected the manuscript. SM wrote the manuscript.

## Conflict of Interest

The authors declare that the research was conducted in the absence of any commercial or financial relationships that could be construed as a potential conflict of interest.
